# Neck circumference and future cardiovascular events in a high-risk population—A prospective cohort study

**DOI:** 10.1186/s12944-016-0218-3

**Published:** 2016-03-05

**Authors:** Yingnan Dai, Xiaojing Wan, Xin Li, Enze Jin, Xueqi Li

**Affiliations:** The Fourth Affiliated Hospital of Harbin Medical University, 37 Yiyuan Street, Habin, 150001 China

## Abstract

**Background:**

The distribution of adipose tissue has been evaluated in relation to cardiovascular risk factors and biochemical components of the metabolic syndrome. Neck circumference (NC) has been shown to have a strong relationship with cardiovascular disease (CVD) and may be a novel indicator of CVD. The aim of this study was to compare the incidence of CVD events in cohorts with different NC distributions, and to correlate NC with future CVD events and relative mortality.

**Methods:**

A prospective cohort study was performed on 12,151 high-risk cardiology outpatients from 2004 until 2014. Anthropometric parameters like body mass index, NC, waist circumference, and hip circumference were measured at baseline and follow-up and compared in different cohorts with high, medium, and low NC. Fatal and non-fatal CVD events were compared in the follow-up study, and survival analysis was conducted. Independent Chi-square tests were performed to compare the incidence of CVD events and mortality among the cohorts and analyze the interactions.

**Results:**

The subjects comprised of 6696 women and 5819 men who completed a mean 8.8-year follow-up. All of the participants had two or more CVD risk factors at baseline. At the end of the study, 4049 CVD events had occurred in 2304 participants. The incidence of non-fatal CVD events was 14.08, 16,65, and 25.21 % in the low-NC, medium-NC, and high-NC cohorts, respectively (*p* < 0.001). The all-cause mortality was 9.77, 11.93, and 19.31 %, and CVD mortality, 4.00, 6.29, and 8.01 %, respectively (*p* < 0.001). Compared with baseline, the number of CVD risk factors in participants had increased from 2.6, 3.0, and 3.4 to 3.5, 4.1, and 4.7 in the low-, medium-, and high-NC cohorts (34, 36, and 38 %), respectively. The event-free survival rate was 95.32, 80.15, and 75.47 %, respectively.

**Conclusions:**

A higher NC indicated a higher incidence of future fatal and non-fatal CVD events and all-cause mortality in both male and female high-risk participants. CVD risk factors increased more in the higher NC group. NC as a novel indicator of CVD showed good predictive ability for CVD events and mortality in a high-risk population.

## Background

Body obesity is associated with an increased risk of developing metabolic syndrome, type 2 diabetes (T2DM), and hypertension and is considered a major risk factor for cardiovascular disease (CVD) events and early mortality. Excessive adiposity is involved in the pathogenesis of coronary heart disease (CHD), since it is closely associated with the development of hypertension, dyslipidemia, and T2DM [1].

In addition to obesity, the distribution of body weight is considered to be an important aspect of metabolic syndrome, and is a stronger predictor of cardiovascular risk. The preferential accumulation of body fat in specific regions is related to disease risk [2]. Generally, visceral and subcutaneous fat are associated with insulin resistance [3]. Visceral adipose tissue is a unique pathogenic fatty deposit that is strongly implicated in the pathogenesis of insulin resistance, T2DM, and ischemic heart disease [4], and closely correlated with the risk of CVD. Due to its correspondence with visceral adipose tissue, waist circumference (WC) is widely used to identify central obesity and is designated as one of the criteria for metabolic syndrome in several clinical definitions. Previous studies have established the relationship between central obesity and the development of CVD [5, 6].

Recent interest is focused on the possible involvement of adiposity as a linking factor in cardiovascular risk. Through direct paracrine effects, locally acting fat depots may contribute to the complications of obesity, in particular vascular disease [7]. Therefore, cardiovascular risk is conferred by specific fat distribution patterns, particularly upper body adiposity, which is as a strong determinant in the population and more strongly associated with glucose intolerance, hyperinsulinemia, diabetes, hypertriglyceridemia, and gout than is lower-body obesity.

Neck circumference (NC), has been evaluated as an index for the distribution of upper-body subcutaneous adipose tissue in relation to cardiovascular risk factors [8] and insulin resistance, and has also been shown to correlate positively with biochemical components of the metabolic syndrome [9]. NC is a valid marker for identifying obese individuals and correlates well with other anthropometric measurements. NC has been independently correlated with cardio-metabolic risk factors above and beyond their relationships with other adiposity measures [10, 11].

However, it is unclear whether NC could predict the incidence of cardiovascular events and mortality. Based on these findings, we hypothesized that NC could predict the incidence of cardiovascular events such as acute coronary syndrome, myocardial infarction, percutaneous coronary intervention, and sudden cardiac death. The aim of this study was to compare the incidence of CVD events in cohorts with different fat distributions and to correlate NC with future CVD events and relative mortality.

## Methods

### Participants

Between February 2004 and December 2006, a total of 18,975 consecutive adults with two or more CVD risk factors who had visited the outpatient clinic of cardiology department in the Fourth Affiliated Hospital of Harbin Medical University were recommended to participate in this study. The definition of CVD risk factors adopted for this study was based on that given by the International Diabetes Federation, which included central obesity based on WC (WC ≥ 85 cm for men; WC ≥ 80 cm for women in the Chinese population), with the addition of at least two of the following four factors: increased triglyceride (TG) (≥1.7 mmol/L), decreased high-density lipoprotein cholesterol (HDL-C) (≤1.03 mmol/L for men and ≤1.29 mmol/L for women), high blood pressure (BP) (SB ≥ 130 or DBP ≥ 85 mmHg), and increased fasting plasma glucose (FPG) (≥5.60 mmol/L) [12]. The Exclusion criteria were as follows: subjects with goiter and other neck masses and deformities; pregnancy; people with severe disabilities or systemic disease, hepatic failure, renal failure; and subjects with diagnosed CVD or T2DM. For all deaths, we sought death certificates, or medical record numbers when appropriate, to understand the cause of death.

After exclusion criteria were applied, 12,151 subjects in total agreed to participate in the longitudinal cohort study, aged 24 to 64 years (47.9 ± 17.7 years, 46.5 % men). The protocol and informed consent documents were approved by the ethics committee of the Fourth Affiliated Hospital of Harbin Medical University. All patients gave written informed consent. Every two years, participants were requested to undergo a clinical examination including blood pressure, blood tests, anthropometric measures, and a self-report medical history review for the mean 8.8 ± 2.1 years of follow-up study.

### Study design

Subjects were categorized on the basis of their NC level, according to the Beijing Community Diabetes Study [13]. The NC categories were as follows: low NC, <33 cm for women and <36 cm for men; medium NC, 33 ≤ NC < 37 cm for women and 36 ≤ NC < 40 cm for men; high NC, ≥37 cm for women and ≥40 cm for men. BMI was categorized on the basis of the following criteria [14]: normal weight, BMI < 23.99; overweight, 24.00 ≤ BMI < 28.00; obese, BMI ≥ 28.00. All subjects underwent a complete medical examination, a clinical consultation, and blood laboratory tests. All individuals provided details of their demographic, medical history, and use of medication at baseline. Subsequent visits were conducted within 6 months of the specified time when subjects were having regular outpatient appointments, were hospitalized because of CVD events, or had made an appointment at the clinic. Anthropometric measures and biomarkers were assessed on each visit.

Cardiovascular events such as acute coronary syndrome, myocardial infarction, percutaneous coronary intervention (performed by clinical doctors), and sudden cardiac death, as well as all-cause death (based on clinical documents) were recorded. Incident cardiovascular morbidity and fatal events were validated by reviewing hospital records and the records of attending physicians, and were classified by an external endpoint committee of cardiologists and doctors. Myocardial infarction was defined as either nonfatal acute myocardial infarction or coronary death. Since it is difficult to perform coronary angiograms on every patient, diagnoses were based on hospital records of symptoms, electrocardiographic signs, enzyme levels (creatine kinase, as well as troponin T or I), and necropsy.

### Anthropometric measures

NC (cm) was measured with the head erect and eyes facing forward, by using a flexible tape positioned horizontally at the upper margin of the laryngeal prominence. Trained doctors or medical students performed the measurement twice. The average of the two results was adopted. Height and body weight were measured with participants standing without shoes and heavy outer garments. Body weight was measured in light clothing to the nearest 0.1 kg, and height to the nearest 0.5 cm. BMI was calculated as weight (in kilograms) divided by height (in meters) squared. WC was the minimum abdominal girth measured to the nearest 0.1 cm. BP was measured by doctors by using a mercury manometer after the subject had rested for at least 5 min. Three measurements were taken, 5 min apart. The mean of the three measurements was used for the analysis. Hypertension was defined as SBP ≥140 mmHg and DBP ≥90 mmHg.

### Biomarkers assessment

Venous blood samples were drawn from all subjects after they fasted overnight (10 h). The blood was transferred into glass tubes and allowed to clot at room temperature. Immediately after clotting, the serum was separated by centrifugation at 3000 rpm for 15 min. Plasma glucose was measured by using the hexokinase glucose-6-phosphate dehydrogenase method. The levels of total cholesterol (TC), TG, HDL-C, and low-density lipoprotein cholesterol (LDL-C) were determined enzymatically by using an auto-analyzer (Type C8000; Roche Ltd, Germany). Hemoglobin A1c (HbA1c) was measured by using high performance liquid chromatography (HPLC; HLC-723G7 hemoglobin HPLC analyzer, Tosoh Corp.) according to the standardized method.

The 1999 World Health Organization diagnostic criteria were used to diagnose diabetes. National Cholesterol Education Programme guidelines were used to define dyslipidemia as follows [15]: hypercholesterolemia, serum cholesterol levels ≥200 mg/dL (≥5.2 mmol/L); hypertriglyceridemia, serum TG levels ≥150 mg/dL (≥1.7 mmol/L); low HDL cholesterol, HDL cholesterol levels <40 mg/dL (<1.04 mmol/L) for men and <50 mg/dL (<1.3 mmol/L) for women; high LDL cholesterol, and LDL cholesterol levels ≥130 mg/dL (≥3.4 mmol/L).

### Statistical analysis

All data were analyzed by using SPSS 17.0 software. All continuous data are presented as mean ± standard deviation (SD) or percentages. Chi-square tests were used to examine differences between categorical data at baseline and follow-up. Changes over the 8.8-year follow-up period were assessed by using repeated measures ANOVA. Data were tested for equality of variance, and a correction for unequal variances was performed when appropriate. All tests of significance were two tailed. Statistical significance was based on a *p* value of <0.05.

## Results

**Fig. 1:**
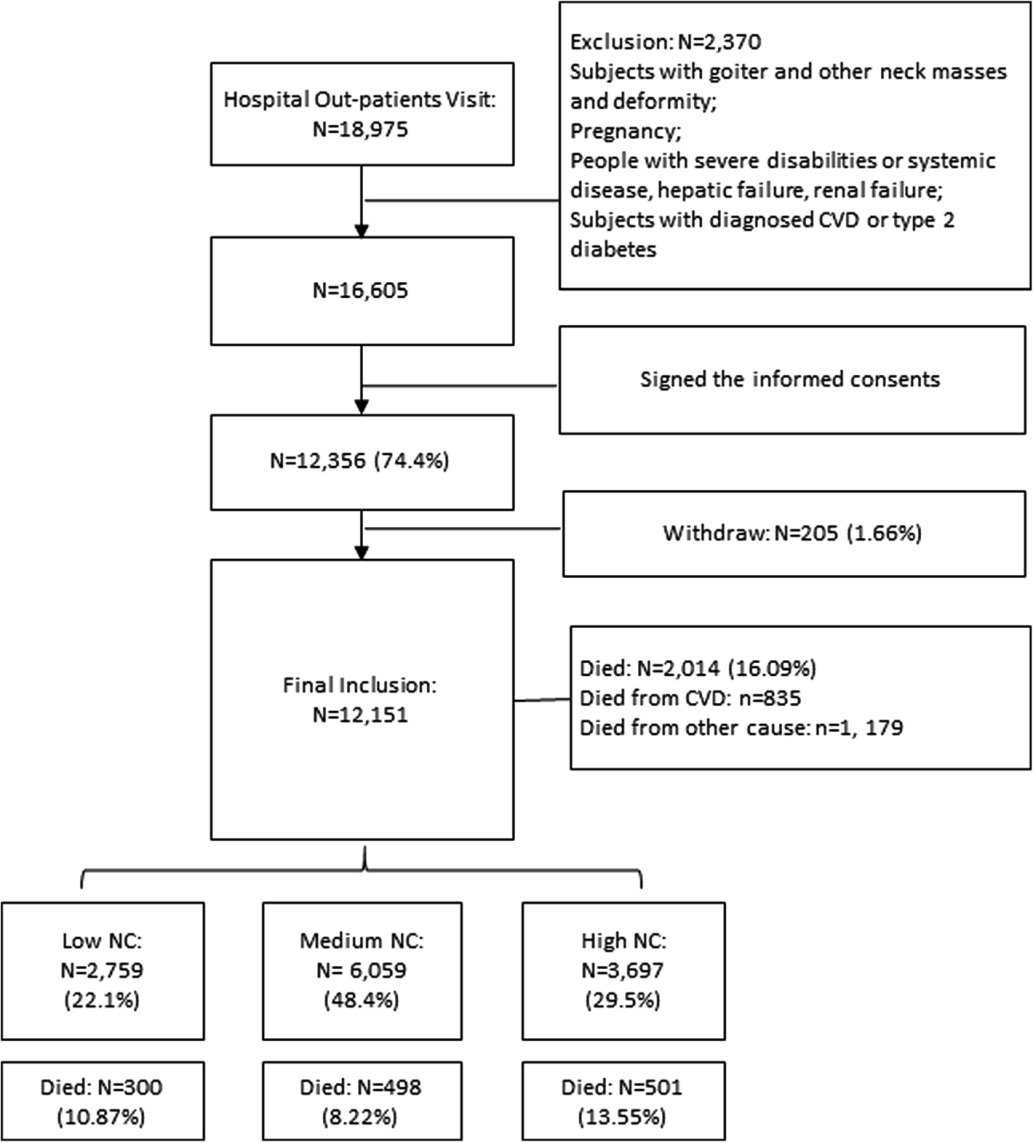
Flow chart of participants’ recruitment

There were 18, 975 subjects who visited the outpatient clinic of the cardiology department of the Fourth Affiliated Hospital of Harbin Medical University in search of help for a cardiovascular problem. After an initial screening, we excluded those whose results did not meet the inclusion criteria (*n* = 2370). Of the patients who were invited to participate in this study, 74.4 % agreed and provided informed consent. During the average 8.8 years of follow-up, 205 subjects withdrew because of migration or loss of contact. Subjects in our study were assigned to one of three cohorts according to their NC levels. There were 2757, 6059, and 3697 participants included in the low, medium, and high NC groups, respectively. At the end of the follow-up study, 10.87, 8.22, and 13.55 % had died from all causes in these three cohorts, respectively (Fig. 1).

Demographic characteristics and CVD risk factors are displayed in Table 1. The study sample consisted of 12,515 individuals, with 6696 (53.50 %) women and 5819 men, a mean age of 47.9 ± 17.7 years, and BMI of 26.31 ± 4.48. All the participants had two or more CVD risk factors. Except for age, HC, WHR, and TG, the other variables were not significantly different between the sexes. Both the sex groups had high levels of FGP, TC, TG, LDL, and a high percentage of smoking and alcohol consumption at baseline. Of the 12,515 subjects, the metabolic syndrome was diagnosed in 2113 (18.66 %), high BP in 4928 (39.38 %), increased FPG in 1872 (14.96 %), increased TG in 4975 (39.75 %), decreased HDL in 3487 (27.86 %), increased LDL in 6711 (53.62 %), and increased TC in 8361 (66.81 %) participants.

**Table 1:** Characteristics of participants at baseline

	Women	Men	*p* value
	*n* = 6696	*n* = 5819	
Age (years)	51.05 ± 15.67	44.29 ± 18.1	0.001
BMI (kg/m^2^)	26.85 ± 5.25	26.09 ± 3.75	NS
NC (cm)	36.31 ± 7.84	38.19 ± 5.94	NS
WC (cm)	84.51 ± 8.56	87.96 ± 12.56	NS
HC (cm)	99.1 ± 8.66	94.66 ± 12.68	<0.001
WHR	0.89 ± 0.08	0.97 ± 0.06	<0.001
FPG (mmol/L)	6.16 ± 1.39	6.86 ± 1.31	NS
HbA1c (%)	5.92 ± 0.67	6.13 ± 0.98	NS
TC (mmol/L)	5.51 ± 1.19	5.29 ± 1.09	NS
TG (mmol/L)	1.79 ± 0.98	2.01 ± 1.19	<0.001
HDL-C (mmol/L)	1.53 ± 0.35	1.33 ± 0.26	NS
LDL-C (mmol/L)	3.51 ± 1.08	3.48 ± 0.88	NS
SBP (mm Hg)	135.71 ± 15.14	133.41 ± 16.84	NS
DBP (mm Hg)	77.66 ± 8.47	78.96 ± 8.65	NS
Smoking (n, %)	2690 (40.17 %)	3547 (60.96 %)	NS
Alcohol drinking (n, %)	893 (13.34 %)	985 (16.93 %)	NS

Values represent mean ± SD, n (%). A *p* value ≤ 0.05 was considered statistically significant. *NS* not significant (*p* > 0.05); *BMI* body mass index; *NC* neck circumference; *WC* waist circumference; *HC* hip circumference; *WHR* waist-hip ratio; *FPG* fasting plasma glucose; *HbA1c* hemoglobin A1c; *TC* total cholesterol; *TG* triglyceride; *HDL-C* high-density lipoprotein cholesterol; *LDL-C* low-density lipoprotein cholesterol; *SBP* systolic blood pressure; *DBP* diastolic blood pressure

At the end of the follow-up study, 4049 CVD events had occurred in 2304 participants. The incidence of non-fatal CVD events was 14.08 % in the low-NC cohort, 16.65 % in the medium-NC cohort, and 25.21 % in the high-NC cohort (*p* < 0.001). The all-cause mortality was 9.77, 11.93, and 19.31 %, and CVD mortality was 4.00, 6.29 and 8.01 %, respectively (*p* < 0.001). After a mean follow-up of 8.8 years (90,605 person-years), we observed 1404 nonfatal CVD events in 859 men and 645 women, and 2014 deaths (835 CVD deaths, 41.5 %) in 1175 men (505 CVD deaths, 43.0 %) and 839 women (430 CVD deaths, 51.0 %).

**Fig. 2:**
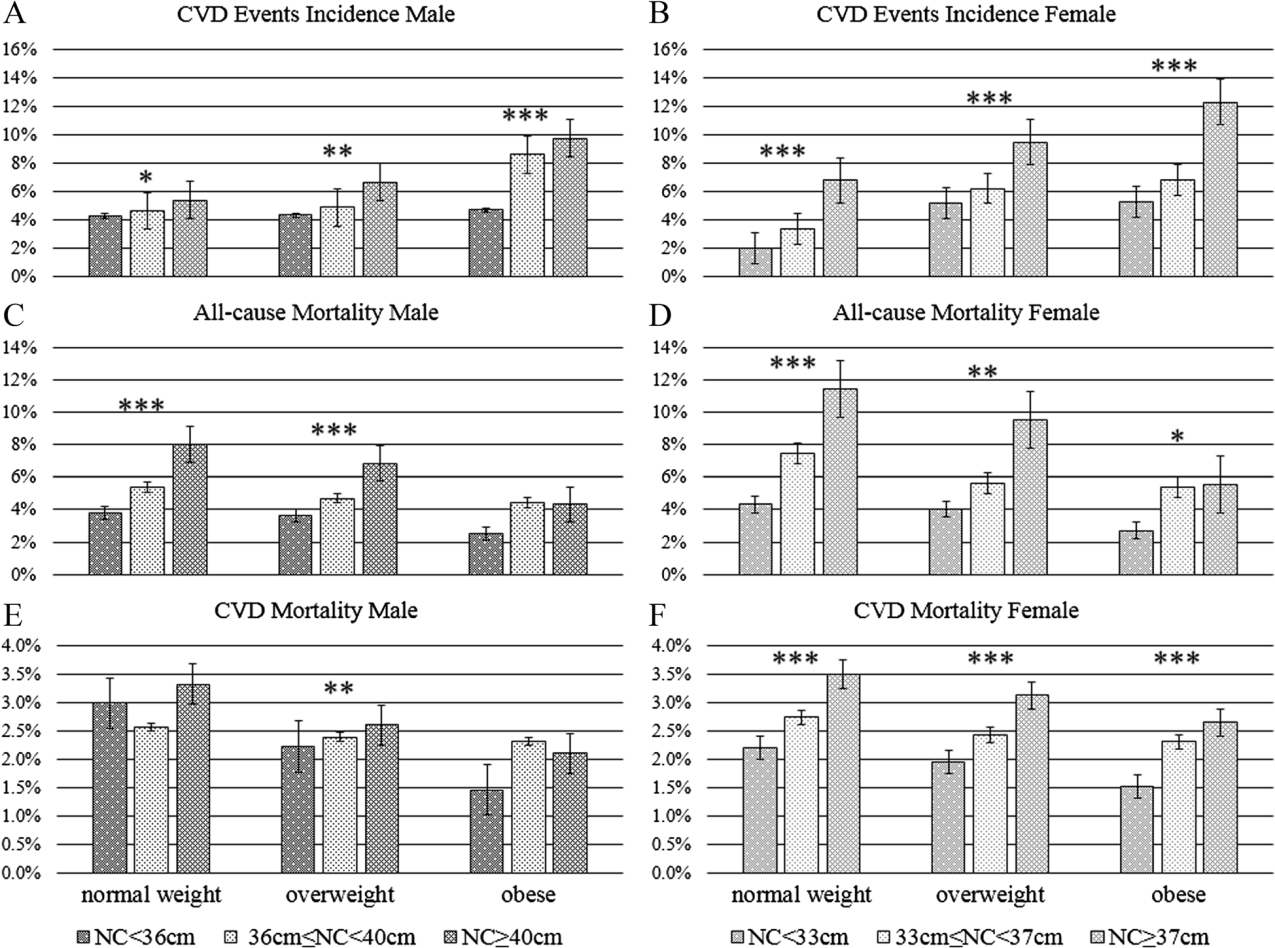
Incidence of CVD events and CVD mortality by NC and BMI. Data are presented as mean ± SEM. P-interaction represents the p value for the interaction between the NC and BMI groups. All means are adjusted for age, smoking, and alcohol use. *p < 0.05; **p < 0.01; ***p < 0.001

When categorized by NC into three groups, an increased risk level for CVD with increased NC was observed (Fig. 2). The interaction between NC and BMI on the incidence of CVD events, all-cause mortality, and CVD mortality were also analyzed. Within each NC group, there was a stepwise increase in the incidence of CVD events, all-cause mortality, and CVD mortality in men and women (*p* < 0.001). Among the men, there was a significant interaction between NC and the incidence of CVD events (*p* < 0.005), all-cause mortality (*p* < 0.001), and CVD mortality (*p* < 0.05). Among the women, a significant interaction was also seen between NC and the incidence of CVD events (*p* < 0.05), all-cause mortality (*p* < 0.001), and CVD mortality (*p* < 0.001). After a mean 8.8-year follow-up, 4049 CVD events had occurred among 2304 participants at baseline, including 1259 women (incidence 18.8 %), and 1045 men (incidence 17.96 %). Despite the presence of CVD events, in all the 12,515 participants at baseline, all-cause mortality at 8.8-year follow-up was 16.09 % (*n* = 2014), including 17.55 % (*n* = 1175) in women and 14.42 % (*n* = 839) in men. There were 935 deaths caused by CVD (mortality 7.47 %), in 505 women (mortality 7.54 %) and 430 men (7.39 %). In the high-NC group, BMI showed a more significant influence on CVD events and mortality. Likewise, in the high BMI group, NC also significantly influenced the CVD outcomes.

Differences in CVD risk levels among NC groups between baseline and follow-up are listed in Table 2. At baseline, significant differences among the NC groups were found for BMI (*p* = 0.05), NC (*p* < 0.001), WC (*p* = 0.001), HC (*p* < 0.001), WHR (*p* < 0.001), FPG (*p* = 0.004), TG (*p* < 0.001), HDL-C (*p* < 0.001), and DBP (*p* = 0.039). In all groups, BMI increased significantly between baseline and follow-up (*p* < 0.001), as well as WC (*p* < 0.001), WHR (*p* < 0.001), FPG (*p* = 0.032), TG (*p* = 0.027), TC (*p* = 0.019), HDL-C (*p* = 0.019), LDL-C (*p* = 0.034), and SBP (*p* = 0.048). However, age, TC, LDL-C, and SBP did not show significant differences among NC groups at baseline (*p* = 0.632, 0.082, 0.116, and 0.137, respectively). NC, HC, and DBP did not change significantly between baseline and follow-up (*p* = 0.051, 0.127, and 0.166, respectively).

**Table 2:** Differences in CVD risk levels among NC groups between baseline and follow-up

	NC < 36 cm		36 cm ≤ NC < 40 cm		NC ≥ 40 cm		*p* values	
	Baseline	Follow-up	Baseline	Follow-up	Baseline	Follow-up	Time	Group
n	*n* = 2759	*n* = 2282	*n* = 6059	*n* = 5254	*n* = 3697	*n* = 2760	-	-
Age (year)	46.33 ± 18.6	56.65 ± 19.12	48.64 ± 17.84	57.42 ± 18.81	47.96 ± 16.58	54.56 ± 16.28	-	0.632
BMI (kg/m^2^)	24.53 ± 5.47	28.4 ± 5.91	26.48 ± 4.75	30.4 ± 3.86	28.18 ± 6.92	30.34 ± 6.12	<0.001	0.05
NC (cm)	31.91 ± 4.2	32.5 ± 4.45	37.67 ± 1.58	37.23 ± 1.73	41.23 ± 1.21	41.55 ± 1.45	0.051	<0.001
WC (cm)	83.26 ± 10.33	84.34 ± 11.64	86.8 ± 9.72	87.54 ± 10.73	87.21 ± 10.96	89.54 ± 12.75	<0.001	0.001
HC (cm)	92.71 ± 10.43	92.53 ± 12.85	96.94 ± 9.22	97.22 ± 9.6	100.55 ± 10.53	103.65 ± 10.35	0.127	<0.001
WHR	0.88 ± 0.14	0.89 ± 0.16	0.92 ± 0.13	0.93 ± 0.15	0.97 ± 0.13	0.99 ± 0.16	<0.001	<0.001
FPG (mmol/L)	6.28 ± 1.33	6.25 ± 1.34	6.46 ± 1.26	6.59 ± 1.22	6.68 ± 1.29	6.77 ± 1.28	0.032	0.004
HbA1c (%)	5.78 ± 0.84	5.98 ± 1.05	5.98 ± 0.67	6.12 ± 0.73	6.25 ± 1.02	6.42 ± 1.35	0.048	0.137
TG (mmol/L)	1.72 ± 0.94	1.78 ± 1.08	1.9 ± 0.91	1.95 ± 0.96	2 ± 0.94	2.15 ± 1.11	0.027	<0.001
TC (mg/dL)	5.17 ± 1.17	5.32 ± 1.01	5.43 ± 1.15	5.67 ± 1.74	5.54 ± 1.67	5.73 ± 1.83	0.019	0.082
HDL-C (mg/dL)	1.67 ± 0.37	1.65 ± 0.27	1.43 ± 0.34	1.25 ± 0.36	1.27 ± 0.29	1.13 ± 0.36	0.006	<0.001
LDL-C (mg/dL)	3.17 ± 0.88	3.26 ± 0.93	3.57 ± 0.95	3.62 ± 1.02	3.61 ± 0.86	3.96 ± 1.01	0.034	0.116
SBP (mm Hg)	133.62 ± 16.78	136.9 ± 15.48	134.63 ± 14.95	137.21 ± 15.17	135.42 ± 14.96	140.07 ± 16.39	0.048	0.137
DBP (mm Hg)	76.27 ± 8.46	76.2 ± 7.7	77.55 ± 8.15	77.34 ± 7.98	80.92 ± 8.95	81.11 ± 9.35	0.166	0.039

Data represent mean ± SD. P interaction is age adjusted. Abbreviations as in Table 1

**Fig. 3:**
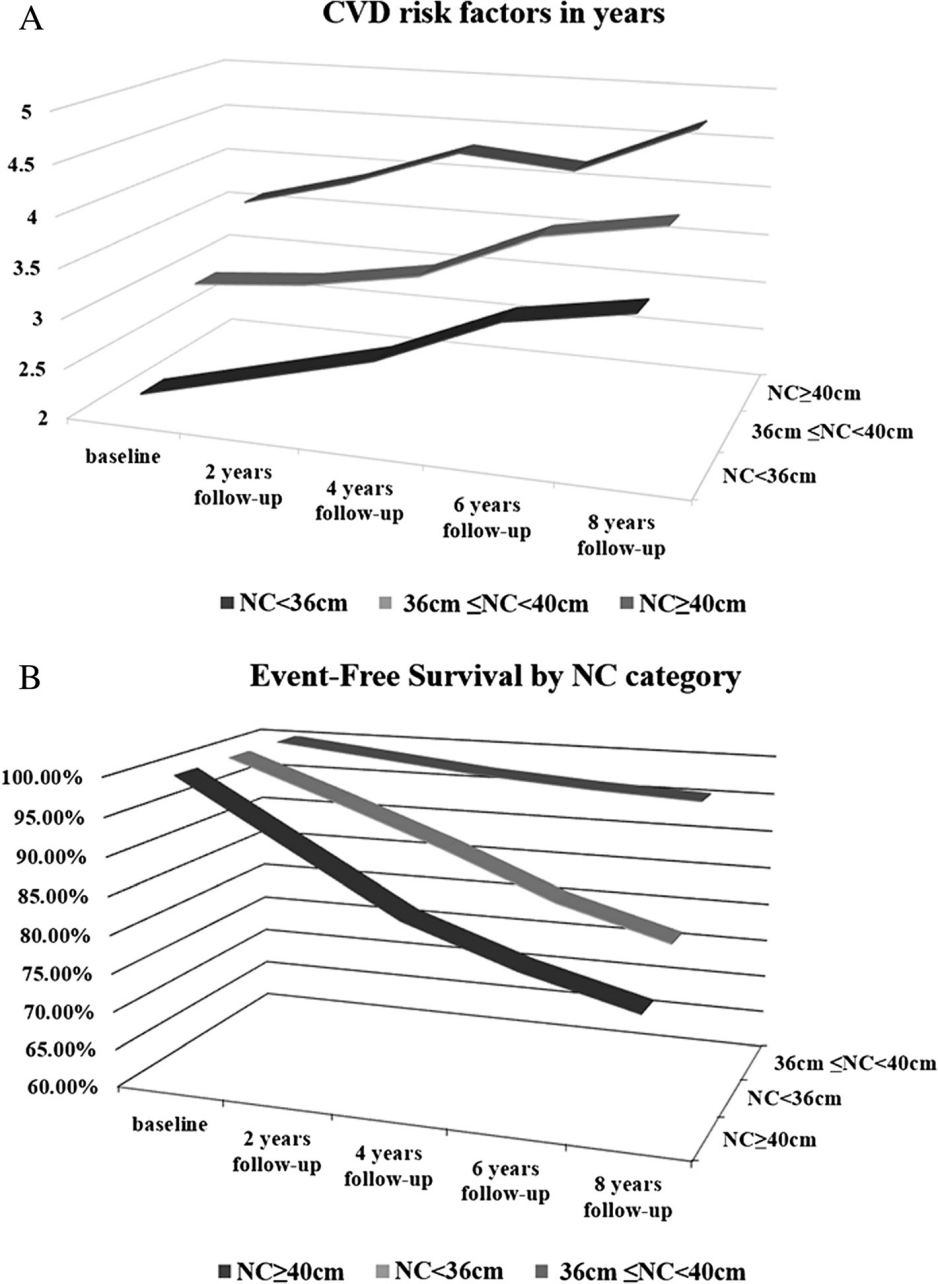
CVD risk factors and event free survival by NC groups. **a** Numbers of CVD risk factors in the different NC groups over time. **b** Event free survival by NC group. Vertical axes show the number of CVD risk factors for each group (**a**) and percentage of event-free survival (**b**) during follow-up. Horizontal axes reflect years of follow-up. The z-axis represents different NC cohorts (described in Methods). Traditional CVD risk factors are described in the Methods

Compared with baseline, the number of CVD risk factors in participants had increased from 2.6, 3.0, and 3.4 to 3.5, 4.1, and 4.7 in the low-, medium-, and high-NC cohorts (34, 36 and 38 %). The event-free survival rate was 95.32, 80.15, and 75.47 %. CVD risk factors and event free survival by NC group in years are shown in Fig. 3. Every 2 years, we calculated the number of CVD risk factors. The CVD risk factors increased in number with NC in the mean 8.8 years of follow-up, with subjects in the high-NC group having 1.2 times as many risk factors as those of participants the low-NC group at the end of follow-up (at baseline, 1.4 times). CVD-event-free survival also decreased in the 8.8 years of follow-up, with subjects in the low-risk group having a 20 % higher CVD-event-free survival rate than that of those in the high NC group (*p* < 0.01).

## Discussion

To the best of our knowledge, this is the first prospective cohort study to evaluate the association between NC and future CVD events and mortality and the first Chinese population cohort to be assessed for an association between fat distribution and CVD mortality. In the present prospective study of a large cohort of Chinese people at high risk of CVD, we examined the incidence of cardiovascular events and mortality in people with different fat distributions (measured by NC and BMI). First, we observed that NC is associated with cardiometabolic risk factors. Second, a higher NC indicated a higher incidence of CVD events in the future. Third, a higher NC was associated with higher all-cause and CVD mortality.

Regional variations in adipocyte secretion have been observed to make differential contributions to disease risk. Subcutaneous fat and peripheral body fat stored predominantly in the femoral, gluteal, and thigh regions help protect against ectopic fat accumulation. Consequently, different accumulations of fat play different roles in obesity-related diseases like insulin resistance, dyslipidemia, and fatty liver. Britton et al. found that visceral adiposity was associated with incident CVD and even cancer [16]. In the Heinz Nixdorf Recall Study, epicardial fat was proved be associated with fatal and nonfatal coronary events, including CVD risk factors and incident myocardial infarction in the general population [17]. More recently, fat accumulation in not only the visceral region but also other regions such as the liver, muscle, pericardial, perivascular, and even perirenal areas was found to be associated with CVD and heart function [18–22].

Free fatty acid release from upper-body subcutaneous fat was reported to be higher than that from lower-body subcutaneous fat [23]. In recent years, several studies have examined the association between NC and cardiometabolic risk factors. One of the most famous among these studies was the Framingham Heart Study, which demonstrated that NC is associated with CVD risk factors even after adjustment for visceral adipose tissue and BMI, and considered NC to be a novel measure of cardiometabolic risk [11]. The effectiveness of NC has been demonstrated several times in the Chinese population. In the Beijing Community Diabetes Study 4, NC was positively associated with the metabolic syndrome in Chinese people with T2DM [13]. In a study by Zhou, NC was significantly correlated with all outcomes of cardio-metabolic risk in both sexes [24]. In the Cardiometabolic Risk in Chinese study of a population of apparently healthy Chinese adults, significant associations were found between high NC and increased risk of insulin resistance and various CVD risk factors. These researchers hold the viewpoint that NC could identify those at high risk of CVD and T2DM [25]. Our results were in line with these previous studies and other studies [9, 26]. In other words, the higher the NC, the greater the risk that cardio-metabolic disturbances will develop in the future. Our findings indicate that NC may be a valid and powerful indicator of CVD.

Although a number of epidemiological studies have demonstrated that diversity in body fat distribution significantly predicts premature death, NC had been used as a novel anthropometric measure of upper-trunk body fat, but had not been assessed for its ability to predict mortality, especially surprising considering its strong associations with CVD risk. The use of BMI combined with measures of central obesity was analyzed recently to assess mortality in subjects with coronary disease [27]. The researchers focused on the role of central obesity in people with normal weight and found that normal weight with central obesity was associated with the highest risk of mortality. However, although NC measurements reduced the shortcomings of WC measurements, which are easily affected by meals, breathing, and clothing, NC was not considered in that study.

This current study is the first prospective study of NC to evaluate its predictive ability for CVD events and mortality. Recent studies have proven the predictive ability of other indicators like WC, HC, and WHR [28–32]; however, the results seemed inconsistent. In the Norfolk cohort, Canoy et al. conducted a population-based prospective study to investigate body fat distribution and the risk of CHD. They compared WC, WHR, and BMI and found that abdominal obesity was more consistently and strongly predictive of CHD than BMI, especially with respect to the risk of obesity-related CHD [33]. WHR, WC, and even BMI were influenced by possible biological factors and confounders. Although directly related to the development of CVD, these indices still confuse clinicians when deciding how they can best predict future events. In our study, NC strongly affected CVD events and mortality, even in the normal BMI group. Because it is not affected by BMI, NC showed a more stable association with CVD.

When the associations with BMI and NC were assessed separately, a higher NC and BMI was associated with a higher incidence of CVD events, but for any given NC, those with a larger BMI had a lower mortality than those with a smaller BMI, regardless of CVD mortality. In the general population, BMI in the overweight and obese ranges has been associated with higher overall mortality [34]. In our findings, BMI remained predictive of CVD and CVD mortality. By contrast, a high BMI was also found to contribute to lower mortality in those with CVD and even in other populations, as observed in many studies [35–37]. Furthermore, BMI has been demonstrated to have a suboptimal correlation with body fat, especially in patients with CVD, because slightly overweight or obese individuals can vary in body fat distribution and associated metabolic risk factors [18, 38]. It suggested to us that some individuals with a high BMI but a low NC may be a typo among our participants, and even those with a low BMI but a high NC. Our results provided more evidence to support the obesity paradox and were consistent with previous conclusions. NC could be a superior predictor of mortality in the population at high-risk of CVD. Compared with BMI, NC is not influenced by these paradoxes and showed a stable ability to predict mortality.

To see the influence of sex on the results, we analyzed the data for both sexes when comparing the interactions of BMI and NC. Both BMI and NC had different impacts on the incidence of CVD events, and also CVD or all-cause mortality in men and women, but to a different degree (Fig. 2). We found increased risks associated with a higher NC value, but with different trends in men and women. A possible explanation for this phenomenon is that there is a greater delivery of free fatty acid from visceral adipose tissue to the liver in women than men [39] and free fatty acid is considered to be one of the most important risk factors for CVD. However, all the interactions were significant for both sexes (*p* < 0.05).

### Strengths and limitations

The main strength of the present study is that we firstly evaluated NC and its association with future CVD outcomes in a high-risk population, with the cohort of individuals manifesting both NC and BMI variability. Furthermore, as this was designed as a prospective study, we succeeded in attaining a high rate of follow-up and minimized the possibility of bias resulting from loss of follow-up. In addition, comparisons between men and women within the same cohort have been scarce or not reported adequately. When comparing the interactions of BMI and NC, we calculated the data for both sexes. Therefore, we could see the influence of sex on the results. The large sample size of the study increased the precision and provided us with extensive information that allowed us to control for potential confounders.

Potential limitations of this study were the lack of comparisons for all anthropometric measures such as WC, HC, and WHR. To consider all these variables together, large-scale population-based studies are needed. A second limitation is the absence of consideration of all the interactions between risk factors like blood pressure or lipids. Although they form part of the causal pathway, taking these factors into account could help toward understanding their role as mediating factors. Further studies are needed to identify the relationship between NC and fatal and nonfatal CVD events in the general population.

## Conclusions

NC is associated with the future incidence of CVD events, mortality, and all-cause mortality, particular among men and women who have risk factors. A graded relationship between NC and CVD events persisted in a population at high risk of CVD. A higher NC indicated a higher incidence of CVD events, mortality, and all-cause mortality in the future. NC could be a superior, simpler, and more consistent indicator of CVD and related mortality in the high-CVD-risk population.

## Abbreviations

BMI: body mass index
BP: blood pressure
CHD: coronary heart disease
CVD: cardiovascular disease
DBP: diastolic blood pressure
FPG: fasting plasma glucose
HbA1c: hemoglobin A1c
HC: hip circumference
HDL-C: high-density lipoprotein cholesterol
HPLC: high performance liquid chromatography
LDL-C: low-density lipoprotein cholesterol
NC: neck circumference
SBP: systolic blood pressure
TC: total cholesterol
TG: triglyceride
T2DM: type 2 diabetes mellitus
WC: waist circumference
WHR: waist-hip ratio
